# Prevalence of intra-abdominal hypertension and markers for associated complications among severe burn patients: a multicenter prospective cohort study (BURNIAH study)

**DOI:** 10.1007/s00068-021-01623-1

**Published:** 2021-03-15

**Authors:** Steven G. Strang, Roelf S. Breederveld, Berry I. Cleffken, Michael H. J. Verhofstad, Oscar J. F. Van Waes, Esther M. M. Van Lieshout

**Affiliations:** 1 Trauma Research Unit, Department of Surgery, Erasmus MC, University Medical Center Rotterdam, P.O. Box 2040, 3000 CA Rotterdam, The Netherlands; 2 Burn Center, Red Cross Hospital, Beverwijk, The Netherlands; 3 Department of Surgery, Leiden University Medical Centre, Leiden, The Netherlands; 4 Burn Center, Maasstad Hospital, Rotterdam, The Netherlands

**Keywords:** Intra-abdominal hypertension, Intra-abdominal pressure, Abdominal compartment syndrome, Surgery, Burn injury

## Abstract

**Purpose:**

Severely burned patients are at risk for intra-abdominal hypertension (IAH) and associated complications such as organ failure, abdominal compartment syndrome (ACS), and death. The aim of this study was to determine the prevalence of IAH among severely burned patients. The secondary aim was to determine the value of urinary intestinal fatty acid binding protein (I-FABP) as early marker for IAH-associated complications.

**Methods:**

A prospective observational study was performed in two burn centers in the Netherlands. Fifty-eight patients with burn injuries ≥ 15% of total body surface area (TBSA) were included. Intra-abdominal pressure (IAP) and urinary I-FABP, measured every 6 h during 72 h. Prevalence of IAH, new organ failure and ACS, and the value of urinary intestinal fatty acid binding protein (I-FABP) as early marker for IAH-associated complications were determined.

**Results:**

Thirty-one (53%) patients developed IAH, 17 (29%) patients developed new organ failure, but no patients developed ACS. Patients had burns of 29% (*P*_25_–*P*_75_ 19–42%) TBSA. Ln-transformed levels of urinary I-FABP and IAP were inversely correlated with an estimate of − 0.06 (95% CI − 0.10 to − 0.02; *p* = 0.002). Maximal urinary I-FABP levels had a fair discriminatory ability for patients with IAH with an area under the ROC curve of 74% (*p* = 0.001). Urinary I-FABP levels had no predictive value for IAH or new organ failure in severe burn patients.

**Conclusions:**

The prevalence of IAH among patients with ≥ 15% TBSA burned was 53%. None of the patients developed ACS. A relevant diagnostic or predictive value of I-FABP levels in identifying patients at risk for IAH-related complications, could not be demonstrated.

**Level of evidence:**

Level III, epidemiologic and diagnostic prospective observational study.

## Introduction

Patients with severe burns are at risk for complications and sequelae resulting in morbidity and death with increasing burn severity. Intra-abdominal hypertension (IAH) and subsequent abdominal compartment syndrome (ACS) are complications in severely burned patients associated with poor outcome. ACS-associated mortality among severe burn patients is estimated at 74.8% [1]. These complications result from the combination of capillary leakage, fluid shifts and hypotension requiring extensive fluid suppletion [2]. The effect of fluid shift is called “third spacing” referring to an extra space to where the fluid shifts to. This space or compartment frequently concerns the abdomen. Possibly, adding up with a circumferential trunk burn which accelerates the process of increasing IAH. Modern resuscitation regimes aim to prevent IAH and ACS. Although the risk has significantly decreased, these complications are still not completely preventable [3, 4]. Early identification of patients at risk for IAH and ACS facilitates decompressive treatment and thereby prevents related morbidity and mortality [5]. To improve this early identification and treatment, epidemiologic knowledge and new diagnostic tools could be helpful.

The current gold standard for IAP determination requires intra-bladder pressure measurements [6]. This is a simple, non-invasive measuring technique, yielding immediate results. However, the level of IAP is neither an indicator for when surgical therapy should be considered, nor is it a reliable indicator for clinical outcome [7]. In this context, Intestinal Fatty Acid-Binding Protein (I-FABP) may be of interest. Evidence is accumulating that the intestines are central in the origin of posttraumatic sequelae [8–10]. Loss of intestinal barrier integrity seems to be an early event in severe illness and trauma that plays a crucial role in the subsequent development of the systemic inflammatory response. The intestinal barrier is maintained by a lining of intestinal epithelial cells (enterocytes) and tight junctions that seal the paracellular space between adjacent enterocytes preventing toxins and bacteria from entering the circulation. Both enterocyte damage and tight junction loss can be triggered by IAH; these can be quantified by levels of I-FABP [11–13]. This marker is rapidly released upon intestinal integrity loss and is easily detectable in urine. Consequently, I-FABP is considered promising for the early identification of intra-abdominal pressure-related complications, they may even be supportive in determining the need for, and timing of decompressive measures to relieve the abdominal pressure.

The primary aim was to determine the prevalence of IAH an ACS in severe burn patients. The secondary aim of the current study was to determine the value of urinary intestinal fatty acid binding protein (I-FABP) levels as early marker for IAH-associated complications.

## Materials and methods

This prospective, observational study was conducted in two burn centers. The study was approved by the Medical Research Ethics Committee (MREC) in the principal study hospital (reference number M012-021) and by the local hospital board in the participating center (reference number L2013-23). Signed informed consent by patient or proxy was obtained.

### Patients (and public involvement statement)

Adult patients (18 years or older) admitted to the burn center between January 1, 2013 and December 31, 2016, with burn wounds (deep dermal and full thickness) with a total body surface area (TBSA) of at least 15% were included. Patients were enrolled as soon as possible, maximally within 48 h after meeting the eligibility criteria. Patients in whom intra-bladder pressure measurement was contra-indicated or unreliable (i.e. patients with a bladder oppressive hematoma or bladder surgery in the past) were excluded from the study. It was not appropriate or possible to involve patients or the public in the design, or conduct, or reporting, or dissemination plans of this research.

### Treatment

Upon identification of IAH or ACS, treatment was initiated in compliance with international guidelines of the WSACS [6]. The IAH/ACS Management Algorithm provides a decision tree for the follow-up of patients related to the IAP level.

### Sample collection

During the first 72 h after enrolment, urine was sampled every 6 h. Samples were taken directly from the catheter tube from a collection point immediately after the indwelling catheter. Urine samples were kept on ice and frozen at − 80 °C within 2 h after collection until further analysis.

### Data collection

Patient characteristics, ICU admission diagnosis, Simplified Acute Physiology Score (SAPS) II, Sequential Organ Failure Assessment score (SOFA, on day 1–4), and Acute Physiology and Chronic Health Evaluation (APACHE) II scores, burn injury characteristics, presence of abdominal and thoracic burns, inhalation injury, intubation status and ventilation settings, hemodynamic parameters, intra-abdominal pressure, diuresis, serum levels of lactate, lactate dehydrogenase (LDH), creatine kinase (CK), pH, base excess, C-reactive protein (CRP), albumin, creatinine, colloid osmotic pressure, administered resuscitation volume (crystalloids and colloids) and administered transfusions, were recorded from the patients’ medical files. The administered volume of crystalloids above the predicted requirement according to Parkland’s formula (4 mL/kg body weight/% TBSA), and the Ivy score (exceeding the ratio of 0.25 L/kg administered volume during study period) were calculated [14].

Urinary concentrations of I-FABP were analyzed in duplicate using a highly specific, commercially available enzyme-linked immunosorbent assay (ELISA) that selectively detects human I-FABP (HyCult Biotechnology, Uden, The Netherlands). I-FABP levels in urine were adjusted to urinary creatinine levels. In contrast with a previous study, serum I-FABP levels were not determined since no advantage over urinary I-FABP was demonstrated [15]. Intra-abdominal pressure was also measured every 6 h during the first 72 h after enrolment. Complications and events, as well as any (secondary) intervention performed such as decompression laparotomy, were recorded. The monitoring of occurrence and extent of new organ failure was based upon change in SOFA score. Furthermore, length of stay in the ICU, and mortality during ICU stay and during hospital stay were registered.

### Outcomes

Intra-abdominal pressure was measured using the intra-bladder technique according to Kron et al. [16] with an instilling volume of 20 mL of sodium chloride 0.9% solution. IAP was measured every 6 h. Intra-abdominal hypertension and abdominal compartment syndrome were diagnosed in compliance with WSACS guidelines. For research purposes, IAH was determined if the average IAP of four consecutive measurements (thus during 24 h) was ≥ 12 mmHg. New organ failure was diagnosed if the score in one of six SOFA subdomains increased to ≥ 3, compared with the day before. ACS was diagnosed if an IAP above 20 mmHg and new organ failure occurred at the same measurement.

### Statistical analysis

An overall population of 100 patients (consisting of 50 with IAH and 50 without) would be sufficient to detect a 0.5 SD increase to 26 ± 10 ng/mL in I-FABP level in patients with IAH (two-sided test with an α level of 0.05) with > 85% statistical power [17]. Statistical analyses were performed using IBM SPSS statistics 22 (IBM, Armonk, NY, USA). Receiver operating characteristic (ROC) analysis and calculation of the Youden index associated criterions were performed with MedCalc Statistical Software version 17.4 (MedCalc Software bvba, Ostend, Belgium; http://www.medcalc.org; 2017).

Normality of continuous data was assessed by the Shapiro Wilk test. Homogeneity of variances was tested using the Levene’s test. Since all continuous data deviated from the standard normal distribution, they are presented as median and quartiles and compared using the Mann–Whitney U-test. Statistical significance was accepted at *p* < 0.05.

A linear mixed-effect model was used to determine the correlation between I-FABP levels and IAP. For this analysis, restricted maximum likelihood method was used, a random intercept and slope were considered. BMI, age, gender, APACHE II score, TBSA, presence of abdominal and thoracic burns, and inhalation injury, resuscitation volume administered in first 8 h and 24 h, intra-abdominal pressure, mean arterial pressure, levels of lactate and CRP, base excess and time from baseline measurement were entered as covariates into the model to evaluate their effect on the correlation between I-FABP level and IAP.

Diagnostic characteristics of urinary I-FABP levels on the determination of IAH and new organ failure were tested using ROC analysis. The corresponding cut-off values and associated sensitivity and specificity were determined. Areas under the ROC curve are shown as measure of overall diagnostic performance.

The predictive value of I-FABP levels on IAH, and organ failure was visualized by plotting re-aligned measurements of the biomarker on the moment (*T* = 0) of peak IAP. Median (*P*_25_–*P*_75_) I-FABP levels were plotted separately for patients with the outcome of interest and control patients.

Since multiple IAP and I-FABP measurements of the same patient are more associated with each other than they are between patients, a multi-level model was used to determine the prognostic role of repeated I-FABP on development of IAH, new organ dysfunction, or ACS. A generalized linear mixed model (GLMM) framework with binomial logit link was chosen for that aim. For this analysis, the binary outcomes of IAH and new organ failure were used as dependent variable. Patient ID was used as a clustering variable as up to 13 measurements per patient were available. BMI, age, gender, APACHE II score, TBSA of burn, presence of abdominal and thoracic burns and inhalation injury, resuscitation volume administered in first 8 h and 24 h, intra-abdominal pressure (only for the organ failure analysis), mean arterial pressure, levels of lactate and CRP, base excess, and time from baseline measurement were entered as covariate into the model to evaluate their effect on the relation between I-FABP level and outcome. Data points that occurred after the event of interest were removed for this analysis. Random intercept and slope were considered. Results were expressed as odds ratios with corresponding 95% confidence interval and *p* values.

## Results

**Fig. 1:**
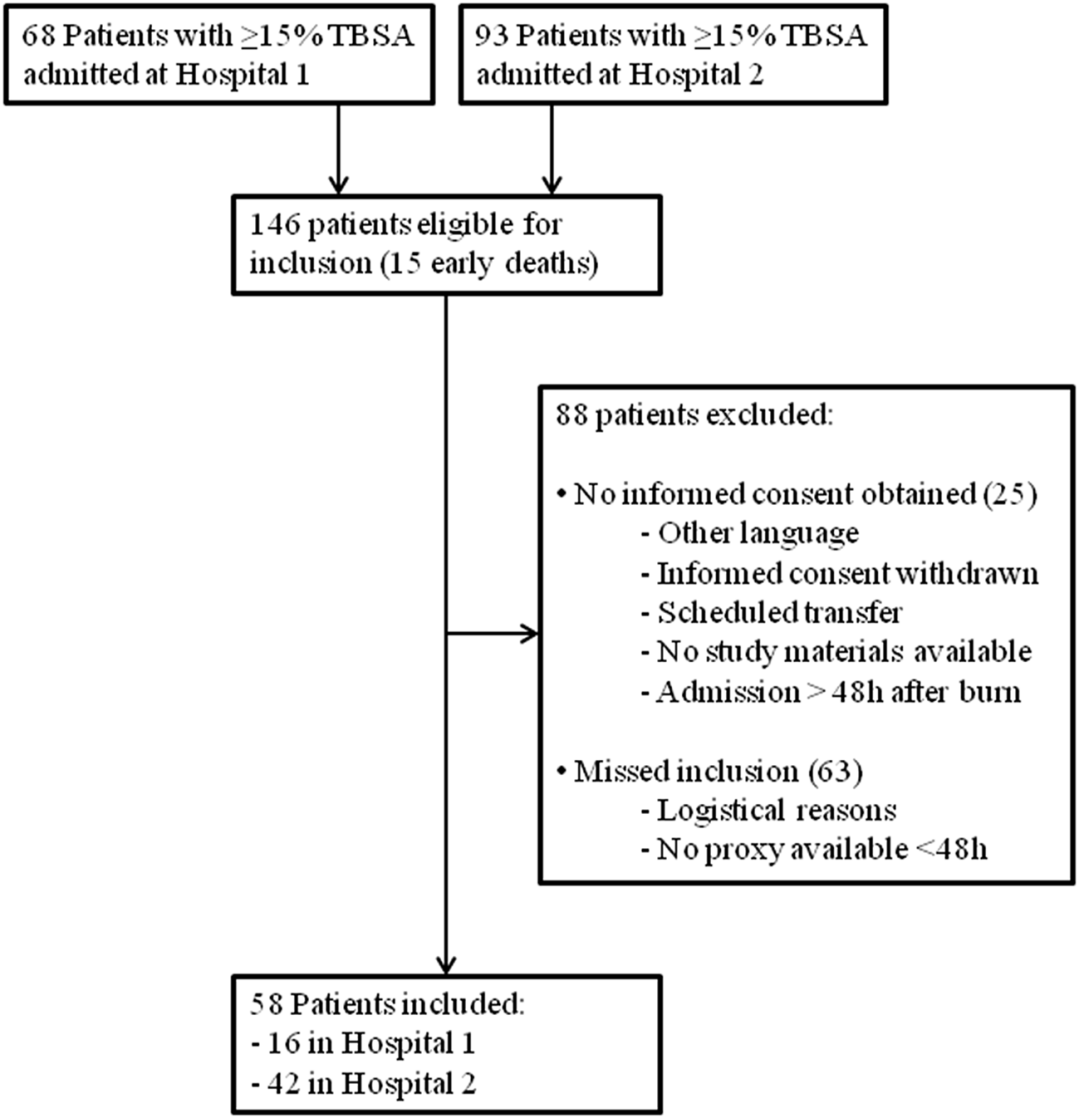
Flowchart

A total of 58 patients were included in two burn centers (Fig. 1). Unexpected difficulties in obtaining informed consent and logistic difficulties resulted in a low inclusion rate. Therefore, the study was ended prematurely before the target number of 100 inclusions was met. No patients were lost to follow-up. Clinical baseline characteristics of 58 included patients are shown in Table 1. Patients had a median age of 48 years (30–58 years), a median APACHE II score of 11 (8–17) and a median burned TBSA of 29% (*P*_25_–*P*_75_ 19–42%, deep dermal and full thickness). Thirty-six (62%) patients were male, 51 (88%) patients had a flame burn, 31(53%) patients developed IAH and 17 (29%) patients developed new organ failure. No patient was diagnosed with ACS (0%, 95% CI 0–0.06). BMI and lactate levels were significantly higher in patients who developed IAH than in patients who did not. Forty (69%) of patients completed the study period of 72 h. Seven (12%) patients died during the study period, 5 (16%) patients had IAH and 2 (7%; *p* = 0.432) had no IAH. The remaining 11 (19%) patients were discharged from the burn ICU and/or had no longer need for a urinary catheter before the 72 h of measurements were completed.

**Table 1:** Baseline characteristics at enrolment for patients with versus without intra-abdominal hypertension

	All	IAH	No IAH	*p* value
	*N* = 58	*N* = 31 (53%)	*N* = 27 (47%)	
Age (years)	48 (30–58)	51 (38–59)	42 (28–54)	0.085
Male	36 (62%)	19 (61%)	17 (63%)	1.000
BMI (kg/m^2^)	26 (23–29)	28 (24–31)	24 (23–27)	0.007
ASA classification				
I	29 (50%)	12 (39%)	17 (63%)	0.214*
II	17 (29%)	11 (36%)	6 (22%)	
III	10 (17%)	6 (19%)	4 (15%)	
IV	2 (3%)	2 (7%)	0 (0%)	
No comorbidity	19 (33%)	8 (26%)	11 (41%)	0.270
APACHE II score	11 (8–17)	12 (8–17)	10 (7–14)	0.223
SAPS II score	26 (22–34)	30 (23–36)	26 (20–31)	0.085
SOFA score	4 (2–6)	4 (2–5)	3 (2–6)	0.937
TBSA (%)	29 (19–42)	21 (18–45)	30 (22–40)	0.235
Burn mechanism				
Flame	51 (88%)	29 (94%)	22 (82%)	0.233
Scald	7 (12%)	2 (6%)	5 (18%)	
Abdominal burns	41 (71%)	21 (68%)	20 (74%)	0.773
Circular abdominal burns	8 (20%)	4 (19%)	4 (20%)	1.000
Inhalation injury	28 (48%)	16 (52%)	12 (44%)	0.610
Crystalloid < 8 h (L)	5.9 (3.9–10.1)	6.0 (4.0–11.0)	5.8 (3.1–9.2)	0.895
Need for vasopressors (%)	35 (60%)	17 (55%)	18 (67%)	0.426
Colloid < 8 h (mL)	106 (63–440)	440 (58–474)	98 (63–131)	0.482
Crystalloid < 24 h (L)	11.7 (8.1–15.8)	13.3 (8.2–15.8)	10.9 (7.7–16.4)	0.691
Colloid < 24 h (mL)	0 (0–0)	0 (0–0)	0 (0–563)	0.616
Ivy Score	211 (148–304)	210 (150–307)	215 (127–289	0.797
Exceeding Parkland formula	34 (59%)	18 (58%)	16 (59%)	1.000
IAP (mmHg)	10 (7–14)	13 (11–15)	6 (4–8)	< 0.001
MAP (mmHg)	82 (72–93)	82 (72–100)	73 (73–90)	0.598
PEEP (cmH_2_O)	7 (5–8)	7 (5–9)	8 (5–8)	0.917
I-FABP urine (pg/nmol creat)	0.1 (0.0–1.4)	0.1 (0.0–1.2)	0.1 (0.0–1.7)	0.749
Lactate (mmol/L)	2.2 (1.5–3.4)	2.6 (1.9–3.8)	2.1 (1.2–2.5)	0.045
Serum creatinine (µmol/L)	69 (55–86)	75 (57–91)	66 (54–85)	0.321
Mortality	7 (12%)	5 (16%)	2 (7%)	0.432

Data are presented as median (*P*_25_–*P*_75_), or number with corresponding percentage (%). *p* values were calculated using a Mann–Whitney *U* test, Fisher exact test or *Pearson Chi Square test.

APACHE, acute physiology and chronic health evaluation; ASA, American Society of Anesthesiologists physical status classification system; BMI, Body Mass Index; Creat, creatinine; IAH, intra-abdominal hypertension; IAP, intra-abdominal pressure; Ivy Score, fluid resuscitation volume during study period per kilogram body weight; MAP, mean arterial pressure; N.D., not determined; PEEP, positive end expiratory pressure; SAPS, Simplified Acute Physiology Score; SOFA, sequential organ failure assessment; TBSA, total body surface area burned

**Fig. 2:**
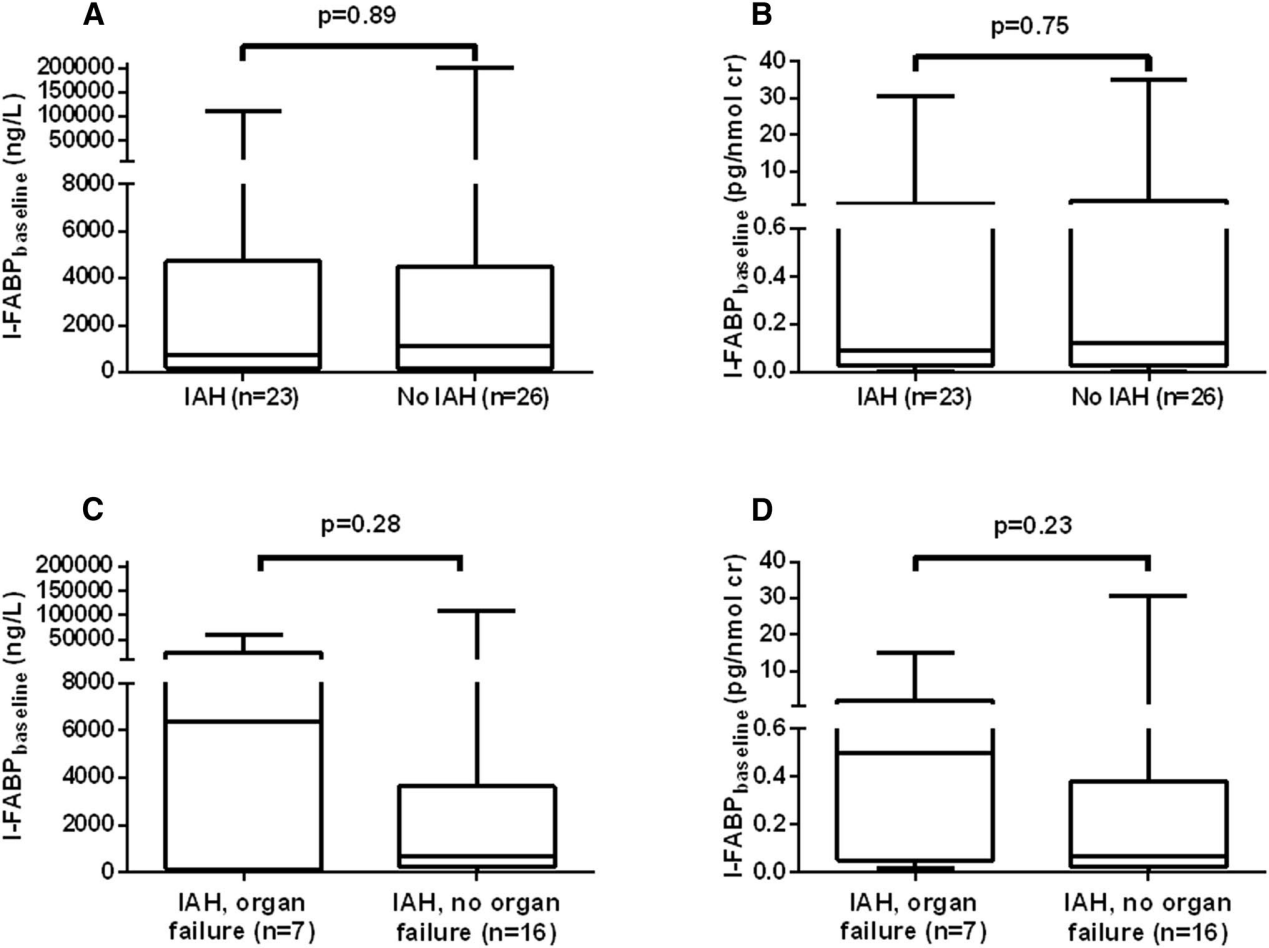
I-FABP levels at baseline in patients who developed IAH (**a**, **b**) or organ failure (**c**, **d**) during study period. Data are shown as median (*P*_25_–*P*_75_) I-FABP levels, uncorrected (**a**, **c**) and corrected (**b**, **d**) for creatinine excretion. Cr, creatinine; IAH, intra-abdominal hypertension; I-FABP, intestinal fatty acid binding protein. *p* values are calculated using Mann–Whitney test

**Fig. 3:**
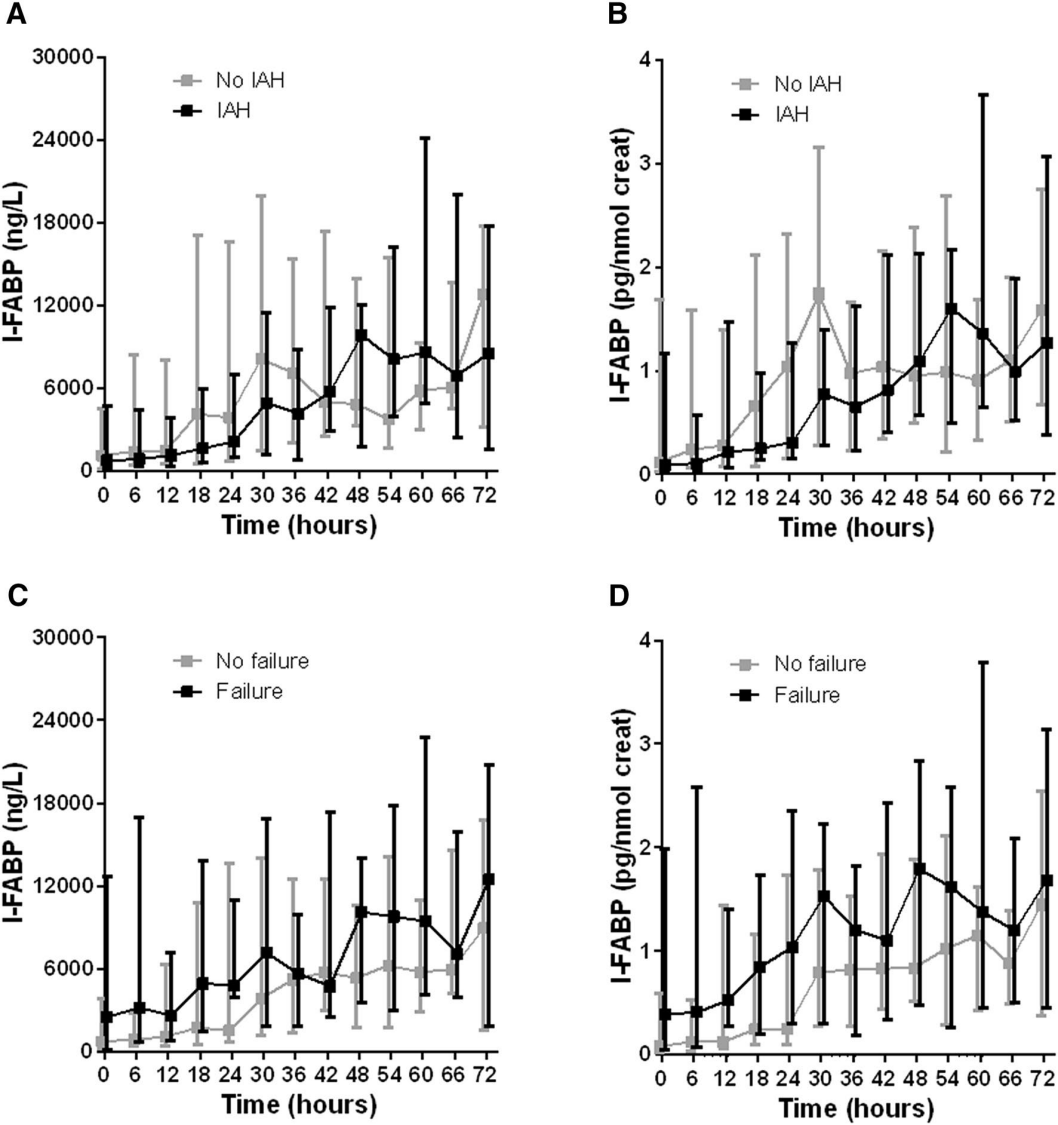
Change in -FABP levels over time in patients with or without intra-abdominal hypertension (**a**, **b**) or new organ failure (**c**, **d**). Data are shown as median (*P*_25_–*P*_75_) I-FABP levels, uncorrected (**a**, **c**) and corrected (**b**, **d**) for creatinine excretion. Creat, creatinine; IAH, intra-abdominal hypertension; I-FABP, intestinal fatty acid-binding protein

The median urinary I-FABP levels (uncorrected and corrected for creatinine excretion) at baseline were not statistically significantly different between groups with or without IAH (Fig. 2). Among patients with IAH, baseline levels of I-FABP also were not different between groups with and without new organ failure during the study period. Median levels of repeated urinary I-FABP measurements (uncorrected, corrected for creatinine excretion, untransformed, as well as logarithmic transformed) in patients with or without IAH and organ failure are shown in Fig. 3. Although all median urinary I-FABP levels (corrected for creatinine excretion) seemed higher in patients who developed new organ failure than in patients who did not, the differences were not statistically significant (Fig. 3d).

**Fig. 4:**
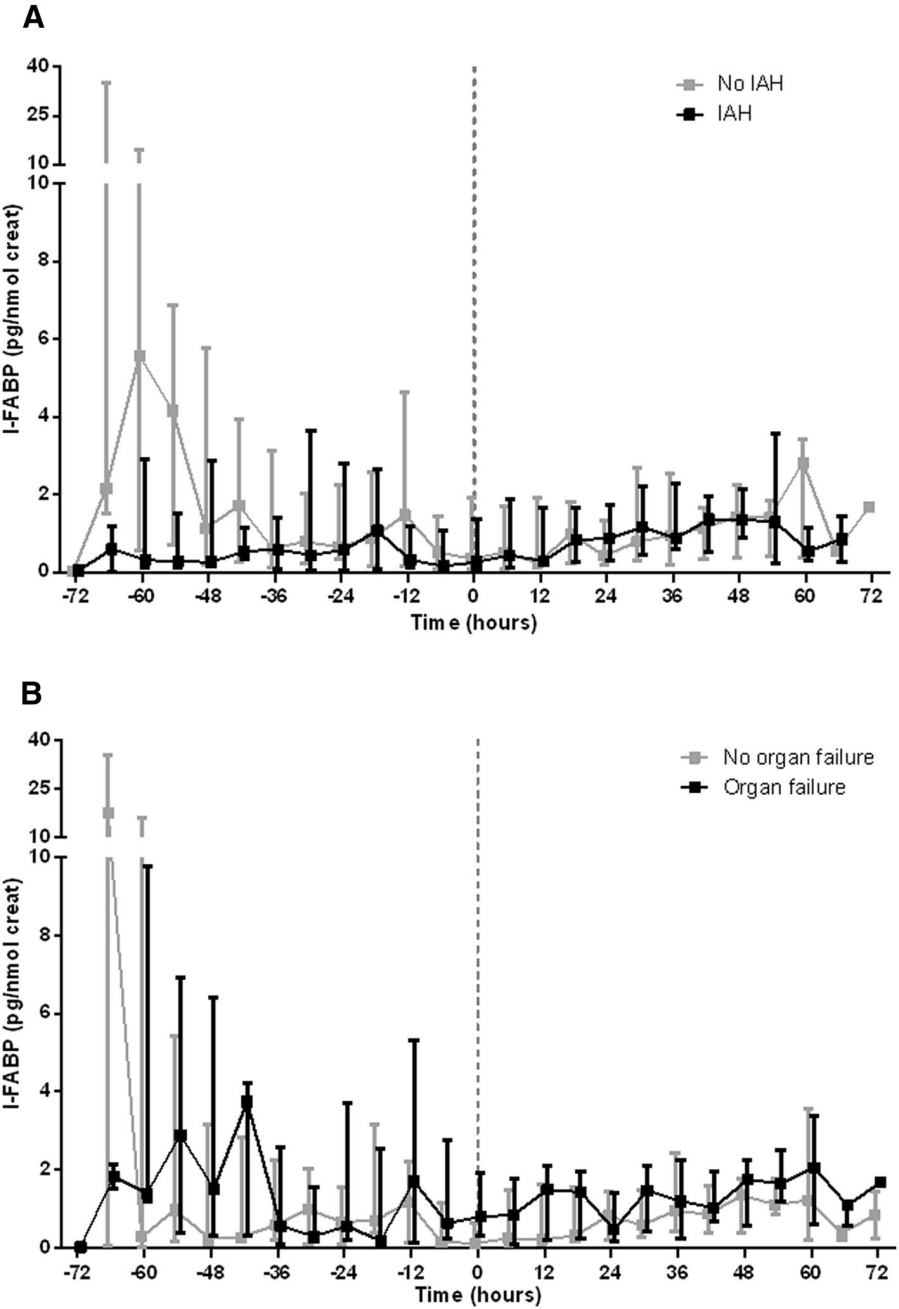
Time course of I-FABP levels before and after peak values of IAP, for patients with or without IAH (**a**) or new organ failure (**b**). Data are represented as median with *P*_25_–*P*_75_. Zero denotes the moment of peak value of IAP. Creat, creatinine; IAH, intra-abdominal hypertension; IAP, intra-abdominal pressure; I-FABP, intestinal fatty acid-binding protein.

Re-aligned measurements of I-FABP (corrected for creatinine excretion; realignment according to peak levels of IAP during study period) demonstrated no statistically significant peaks prior to the peak value of IAP (Fig. 4). Similarly as described above, the median I-FABP levels were consistently higher in patients with new organ failure than in patients without, but not statistically significant.

Univariate correlation analysis between intra-abdominal pressure and I-FABP level showed an unexpected inverse correlation of IAP with both uncorrected and corrected Ln-transformed urinary I-FABP levels, with an estimate of − 0.06 (*p* = 0.001 and *p* = 0.002, respectively). This effect was more pronounced in the subgroup analysis of patients with IAH; here the correlation estimates within the subgroup were − 0.08 (*p* < 0.001) and − 0.09 (*p* < 0.001) for uncorrected and corrected Ln-transformed urinary I-FABP, respectively (Table 2). Multivariable analysis confirmed statistically significant inverse correlation for corrected, Ln-transformed urinary I-FABP levels (i.e., corrected for creatinine excretion; *p* = 0.048 for all patients and *p* = 0.043 for patients with IAH) and IAP.

**Table 2:** Univariate and multivariable correlation between I-FABP level and intra-abdominal pressure

Untransformed I-FABP						
	Urinary I-FABP (ng/L)			Urinary I-FABP (pg/nmol creat)		
Unadjusted linear mixed model	*N*	Estimate (95% CI)	*p*	*N*	Estimate (95% CI)	*p*
All patients	479	− 275.25 (− 587.60 to 37.09)	0.084	477	− 0.02 (− 0.10 to 0.06)	0.557
Patients with IAH	242	− 290.52 (− 562.85 to − 18.18)	**0.037**	240	− 0.03 (− 0.13 to 0.07)	0.583

Adjusted linear mixed model	*N*	Estimate (95% CI)	*p*	*N*	Estimate (95% CI)	*p*
All patients	124^A^	− 243.83 (− 775.14 to 287.48)	0.338	123^A^	0.16 (− 0.14 to 0.45)	0.285
Patients with IAH	72^B^	− 360.18 (− 851.07 to 130.71)	0.147	71^B^	0.002 (− 0.23 to 0.23)	0.983

Ln-transformed I-FABP						
	Urinary I-FABP (ng/L) (Ln)			Urinary I-FABP (pg/nmol creat) (Ln)		
Unadjusted linear mixed model	*N*	Estimate (95% CI)	*p*	*N*	Estimate (95% CI)	*p*
All patients	478	− 0.06 (− 0.10 to − 0.03)	**0.001**	477	− 0.06 (− 0.10 to − 0.02)	**0.002**
Patients with IAH	241	− 0.08 (− 0.13 to − 0.04)	**< 0.001**	240	− 0.09 (− 0.14 to − 0.05)	**< 0.001**

Adjusted linear mixed model	*N*	Estimate (95% CI)	*p*	*N*	Estimate (95% CI)	*p*
All patients	167^C^	− 0.05 (− 0.10 to 0.00)	0.077	166^E^	− 0.05 (− 0.10 to 0.00)	**0.048**
Patients with IAH	72^D^	− 0.04 (− 0.12 to 0.05)	0.389	84^E^	− 0.06 (− 0.11 to − 0.00)	**0.043**

The coefficient estimates for the linear mixed models are shown with 95% confidence interval between brackets

CI, confidence interval; creat, creatinine; CRP, C-Reactive Protein; IAH, intra-abdominal hypertension; I-FABP, intestinal fatty acid binding protein; *N*, number of samples used for this analysis, i.e., this number includes multiple observations of same patients.

^A^Adjusted for lactate level and TBSA

^B^Adjusted for BMI, lactate level and SOFA score at baseline

^C^Adjusted for CRP and resuscitation volume of crystalloids given in first 8 h

^D^Adjusted for lactate and resuscitation volume of crystalloids given in first 8 h

^E^Adjusted for CRP, MAP, and resuscitation volume of crystalloids given in first 8 h

The maximal I-FABP levels per patients showed to be indicative for IAH with a sensitivity of 75% and a specificity of 70% for uncorrected I-FABP levels and a sensitivity of 75% and specificity of 74% for I-FABP levels corrected for creatinine excretion. The overall accuracy was fair with an area under ROC curve of both 0.74 (*p* = 0.001 and *p* = 0.002, respectively; Table 3). Other expressions of urinary I-FABP showed no discriminatory ability between patients with or without IAH or organ failure.

**Table 3:** Diagnostic performance of I-FABP levels for intra-abdominal hypertension and organ failure

		Event	*N*	AUC (95% CI)	*P*	Cut-off value (95% CI)	Sensitivity (%) (95% CI)	Specificity (%) (95% CI)
*All patients*								
Maximum I-FABP	(ng/L)	IAH	51	0.74 (0.60–0.85)	**0.001**	8,337 (1,385–22,672)	75 (53–90)	70 (50–86)
	(pg/nmol)		51	0.74 (0.59–0.85)	**0.002**	1.16 (0.06–1.99)	75 (53–90)	74 (54–89)
Median I-FABP	(ng/L)	IAH	51	0.61 (0.47–0.75)	0.185	1,712 (770–22,311)	67 (45–84)	67 (46–84)
	(pg/nmol)		51	0.64 (0.49–0.77)	0.102	0.52 (0.11–3.64)	75 (53–90)	59 (39–78)
Maximum I-FABP	(ng/L)	Organ failure	54	0.55 (0.41–0.68)	0.571	24,009 (15,098–60,396)	81 (54–96)	34 (20–51)
	(pg/nmol)		54	0.52 (0.38–0.66)	0.821	2.89 (1.86–35.27)	75 (48–93)	42 (26–59)
Median I-FABP	(ng/L)	Organ failure	54	0.54 (0.40–0.68)	0.598	2,939 (651–11,001)	69 (41–89)	55 (38–71)
	(pg/nmol)		54	0.55 (0.41–0.69)	0.545	0.87 (0.20–3.64)	44 (20–70)	71 (54–85)
*Patients with IAH*								
Maximum I-FABP	(ng/L)	Organ failure	27	0.55 (0.35–0.74)	0.705	12,900 (265–27,951)	63 (25–92)	63 (38–84)
	(pg/nmol)		27	0.54 (0.34–0.73)	0.763	1.58 (0.06–2.89)	75 (35–97)	53 (29–76)
Median I-FABP	(ng/L)	Organ failure	27	0.63 (0.43–0.81)	0.334	2,939 (196–8,525)	75 (35–97)	63 (38–84)
	(pg/nmol)		27	0.60 (0.39–0.78)	0.462	0.87 (0.29–1.69)	50 (16–84)	79 (54–94)

Data were analyzed using ROC analysis. Sensitivity and specificity are shown as percentage. AUC, cut-off value, sensitivity, and specificity are shown with 95% confidence interval within brackets

ACS, abdominal compartment syndrome; AUC, area under the receiver operating characteristics curve; CI, confidence interval; creat, creatinine; IAH, intra-abdominal hypertension; IAP, intra-abdominal pressure; I-FABP, intestinal fatty acid binding protein; *N*, number of patients in the analysis; ROC, receiver operating characteristic

Unadjusted generalized linear mixed model analysis demonstrated no statistically significantly predictive value of repeated I-FABP measurements on the development of IAH or new organ failure (Table 4). Adjusting for covariates did not improve any of the prediction models in this study.

**Table 4:** Prognostic role of I-FABP levels on development of intra-abdominal hypertension, abdominal compartment syndrome, and organ failure

		Event	Unadjusted analysis			Adjusted analysis		
			*N*	OR (95% CI)	*p*	*N*	OR (95% CI)	*p*
*All patients*								
Urinary I-FABP	(ng/L)	IAH	333	1.000 (1.000–1.000)	0.884	321	1.000 (1.000–1.000)^A^	0.794
	(pg/nmol creat)		333	1.048 (0.907–1.211)	0.525	321	1.015 (0.853–1.207)^A^	0.869
	(ng/L) (Ln-transformed)		333	0.845 (0.569–1.257)	0.405	321	0.806 (0.524–1.238)^A^	0.323
	(pg/nmol creat) (Ln-transformed)		333	0.832 (0.567–1.220)	0.346	321	0.801 (0.529–1.212)^A^	0.292
Urinary I-FABP	(ng/L)	Organ failure	457	1.000 (1.000–1.000)	0.907	457	1.000 (1.000–1.000)^B^	0.898
	(pg/nmol creat)		455	1.012 (0.835–1.225)	0.906	455	0.991 (0.696–1.411)^B^	0.961
	(ng/L) (Ln-transformed)		456	1.093 (0.699–1.709)	0.697	456	1.041 (0.566–1.913)^B^	0.897
	(pg/nmol creat) (Ln-transformed)		455	1.112 (0.711–7.139)	0.641	455	1.010 (0.554–1.842)^B^	0.975
*Patients with IAH*								
Urinary I-FABP	(ng/L)	Organ failure	223	1.000 (1.000–1.000)	0.723	223	1.000 (1.000–1.000)^C^	0.723
	(pg/nmol creat)		221	1.030 (0.800–1.328)	0.817	221	1.030 (0.800–1.328)^C^	0.817
	(ng/L) (Ln-transformed)		222	1.161 (0.603–2.238)	0.654	222	1.161 (0.603–2.238)^C^	0.654
	(pg/nmol creat) (Ln-transformed)		221	1.133 (0.588–2.185)	0.707	221	1.133 (0.588–2.185)^C^	0.707

Data were analyzed using a generalized linear mixed model. The OR is shown with 95% confidence interval within brackets

ACS, abdominal compartment syndrome; CI, confidence interval; creat, creatinine; IAH, intra-abdominal hypertension; I-FABP, intestinal fatty acid binding protein; OR, odds ratio.

^A^Adjusted for BMI

^B^Adjusted for inhalation, TBSA and Resuscitation volume of crystalloids < 24 h

^C^Adjusting for covariates did not significantly improve the model, therefore unadjusted OR with corresponding 95% confidence intervals and *p* values are shown

## Discussion

The prevalence of intra-abdominal hypertension in patients ≥ 15% TBSA burned was 53%. None of these patients developed abdominal compartment syndrome. This study also showed that urinary I -FABP levels have no significant diagnostic or predictive value for IAH and related organ failure in this population.

The prevalence of IAH in severely burned patients as found in this study is comparable with findings from previous studies. A systematic review from 2013 reported a pooled prevalence of 64.7–74.5% in severely burned patients with a various cut-off value of TBSA injured [1]. A more recent African study reported an IAH-prevalence of 57.8% in a group of 64 adults and children with a TBSA of 25% and 20%, respectively [4]. However, the absence of ACS cases in the current study contrasts literature. An observational study included 56 mechanical ventilated burn patients between 2007 and 2009 and reported that 16 patients developed ACS (29%). It is known that reduction of crystalloid resuscitation volume, early use of albumin and vasopressors reduces the risk of ACS [18]. Reduction of IAH and ACS prevalence is confirmed in several studies in burn and other populations and is uniformly attributed to modern restrained resuscitation regimes [19–22]. The present study is the first and largest epidemiological study of severe burn patients which reports no cases of ACS. Most likely, the awareness of IAP-related problems, careful IAP monitoring and restrictive resuscitation regimes resulted in absence of ACS cases in this series. The data of the present study do not support a causal relation between restrictive fluid resuscitation and absence of ACS cases. Patients with and without IAH received comparable volumes of crystalloids and had comparable injuries. However, adding administered volume of crystalloids in the multivariable correlation between Ln-transformed I-FABP and IAP, improved the model. Indirectly, it could be argued that the administered volume of crystalloids and IAP are correlated. One might wonder whether routine IAP measurements are useful at the current low prevalence of ACS. Perhaps it is better to only perform IAP measurements in patients who develop organ failure. In case of increased IAP, ACS can be included in the differential diagnosis as cause of organ failure.

The value of urinary intestinal fatty acid binding protein (I-FABP) levels as early marker for IAH-associated complications was limited in this study. I-FABP levels were hypothesized as indicative for imminent ACS, subsequent early treatment for that aim would benefit outcome. As no cases of ACS were present, this hypothesis could not be tested. Nevertheless, an inverse correlation between IAP and I-FABP levels was demonstrated. However, the inverse correlation between IAP and I-FABP is contradictory with the expected outcome, as reduced intestinal blood flow resulting from increased IAP is expected to cause mucosal ischemia which proved to be related to increased levels of I-FABP [23–26]. This was confirmed by recently published outcomes of the I-Fabulous study [15]. That study demonstrated a positive correlation between IAP and I-FABP among 198 ICU patients with two or more risk factors for IAH. Since IAP seems correlated to resuscitation volume, it is possible that the enterocyte damage as measured by I-FABP, resulted from under resuscitation in patients with low IAPs. This is, however, somewhat contradicted by the higher lactate levels (which are also increased with under resuscitation) in patients with IAH. I-FABP showed to be a sensitive but non-specific marker for enterocyte damage, but this shows that it remains a bit unclear what exactly is being measured with it in this study.

This study has several limitations. First, the study was ended prematurely as it was difficult to obtain informed consent of these severely injured patients despite the staged informed consent procedure. Upon admission, informed consent was obtained from a legal representative. Patients signed informed consent themselves if they were capable. In most cases, the patients were not capable. The legal representatives frequently did not consider themselves capable of providing the informed consent, or they did not see the added value for the patient in it. Therefore, several patients were not included. After a period of 48 months, 58 patients were included without one patient diagnosed with abdominal compartment syndrome. It was not considered useful to continue the inclusion process. Therefore, the study is underpowered. It seems that ACS only emerges in a more severely burned patient group. Future studies into ACS in patients with severe burns would preferably use a higher % TBSA as inclusion criterion. Second, this series consists of non-consecutive patients with a TBSA ≥ 15%. For epidemiological purposes, a consecutive series of patients would be preferred. The non-consecutive series was due to unexpected difficulties in informed consent procedure. The problem of the difficult inclusion also arose with the large number of different doctors who obtained the informed consent. Ideally, this number would be very limited to preferably one. The question is whether the research question justifies a larger study in an already heavily studied patient group. Lastly, for clinical purposes, the definition of IAH, ACS, and organ failure are very clear. It, however, remains challenging to relate the definition of organ failure to that of ACS. Organ failure is best determined using the SOFA score, but this is normally determined only once a day. In light of very rapidly developing abdominal compartment syndrome (a few hours), this is not ideal. Diagnosing ACS, and specifically at what moment ACS emerged, remains quite subjective. The hypothesis that the I-FABP biomarker could provide a more objective measure for this, could not be demonstrated. Beside I-FAPB, D-lactate and ischemia modified albumin (IMA) may be of interest for future research with that aim [27].

## Conclusion

The prevalence of IAH among severely burned patients with a TBSA ≥ 15% is 53%, none of the patients developed abdominal compartment syndrome. Data could not show a relevant diagnostic or predictive value of I-FABP levels in identifying patients at risk for intra-abdominal pressure-related complications.

## Data Availability

Data can be accessed on request at the Trauma Research Unit, Department of Surgery, Erasmus MC, University Medical Center, Rotterdam, the Netherlands.

## References

[1] Strang SG, Van Lieshout EMM, Breederveld RS, Van Waes OJF (2014). A systematic review on intra-abdominal pressure in severely burned patients. Burns.

[2] Evers LH, Bhavsar D, Mailander P (2010). The biology of burn injury. Exp Dermatol.

[3] Bjorck M, Petersson U, Bjarnason T, Cheatham ML (2011). Intra-abdominal hypertension and abdominal compartment syndrome in nontrauma surgical patients. Am Surg.

[4] Mbiine R, Alenyo R, Kobusingye O, Kuteesa J, Nakanwagi C, Lekuya HM, Kituuka O, Galukande M (2017). Intra-abdominal hypertension in severe burns: prevalence, incidence and mortality in a sub-Saharan African hospital. Int J Burns Trauma.

[5] Ramirez JI, Sen S, Palmieri TL, Greenhalgh DG (2018). Timing of laparotomy and closure in burn patients with abdominal compartment syndrome: effects on survival. J Am Coll Surg.

[6] Kirkpatrick AW, Roberts DJ, De Waele J, Jaeschke R, Malbrain ML, De Keulenaer B, Duchesne J, Bjorck M, Leppaniemi A, Ejike JC (2013). Intra-abdominal hypertension and the abdominal compartment syndrome: updated consensus definitions and clinical practice guidelines from the World Society of the Abdominal Compartment Syndrome. Intensive Care Med.

[7] Reintam Blaser A, Regli A, De Keulenaer B, Kimball EJ, Starkopf L, Davis WA, Greiffenstein P, Starkopf J, Incidence RF (2019). Outcomes of Intra-Abdominal Study I. Incidence, risk factors, and outcomes of intra-abdominal hypertension in critically ill patients—a prospective multicenter study (IROI Study). Crit Care Med..

[8] Fink MP, Delude RL (2005). Epithelial barrier dysfunction: a unifying theme to explain the pathogenesis of multiple organ dysfunction at the cellular level. Crit Care Clin.

[9] Moore FA (1999). The role of the gastrointestinal tract in postinjury multiple organ failure. Am J Surg.

[10] Rotstein OD (2000). Pathogenesis of multiple organ dysfunction syndrome: gut origin, protection, and decontamination. Surg Infect (Larchmt)..

[11] de Haan JJ, Lubbers T, Derikx JP, Relja B, Henrich D, Greve JW, Marzi I, Buurman WA (2009). Rapid development of intestinal cell damage following severe trauma: a prospective observational cohort study. Crit Care.

[12] Gollin G, Marks C, Marks WH (1993). Intestinal fatty acid binding protein in serum and urine reflects early ischemic injury to the small bowel. Surgery.

[13] Pelsers MM, Hermens WT, Glatz JF (2005). Fatty acid-binding proteins as plasma markers of tissue injury. Clin Chim Acta.

[14] Ivy ME, Atweh NA, Palmer J, Possenti PP, Pineau M, D'Aiuto M (2000). Intra-abdominal hypertension and abdominal compartment syndrome in burn patients. J Trauma.

[15] Strang SG, Habes QLM, Van der Hoven B, Tuinebreijer WE, Verhofstad MHJ, Pickkers P, Van Lieshout EMM, Van Waes OJF. Intestinal fatty acid binding protein as a predictor for intra-abdominal pressure-related complications in patients admitted to the intensive care unit; a prospective cohort study (I-Fabulous study). J Crit Care. 2020. 10.1016/j.jcrc.2020.08.023. 10.1016/j.jcrc.2020.08.023 32980233

[16] Kron IL, Harman PK, Nolan SP (1984). The measurement of intra-abdominal pressure as a criterion for abdominal re-exploration. Ann Surg.

[17] Kanda T, Fujii H, Tani T, Murakami H, Suda T, Sakai Y, Ono T, Hatakeyama K (1996). Intestinal fatty acid-binding protein is a useful diagnostic marker for mesenteric infarction in humans. Gastroenterology.

[18] Park SH, Hemmila MR, Wahl WL (2012). Early albumin use improves mortality in difficult to resuscitate burn patients. J Trauma Acute Care Surg.

[19] Ruiz-Castilla M, Barret JP, Sanz D, Aguilera J, Serracanta J, Garcia V, Collado JM (2014). Analysis of intra-abdominal hypertension in severe burned patients: the Vall d'Hebron experience. Burns.

[20] Sanchez-Sanchez M, Garcia-de-Lorenzo A, Herrero E, Asensio MJ, Galvan B, Cachafeiro L (2014). Prevalence of intra-abdominal hypertension (IAH) among patients with severe burns. Burns.

[21] Balogh ZJ, Lumsdaine W, Moore EE, Moore FA (2014). Postinjury abdominal compartment syndrome: from recognition to prevention. Lancet.

[22] Balogh ZJ, Martin A, van Wessem KP, King KL, Mackay P, Havill K (2011). Mission to eliminate postinjury abdominal compartment syndrome. Arch Surg.

[23] Kanda T, Tsukahara A, Ueki K, Sakai Y, Tani T, Nishimura A, Yamazaki T, Tamiya Y, Tada T, Hirota M (2011). Diagnosis of ischemic small bowel disease by measurement of serum intestinal fatty acid-binding protein in patients with acute abdomen: a multicenter, observer-blinded validation study. J Gastroenterol.

[24] Sun DL, Cen YY, Li SM, Li WM, Lu QP, Xu PY (2016). Accuracy of the serum intestinal fatty-acid-binding protein for diagnosis of acute intestinal ischemia: a meta-analysis. Sci Rep.

[25] Thuijls G, van Wijck K, Grootjans J, Derikx JP, van Bijnen AA, Heineman E, Dejong CH, Buurman WA, Poeze M (2011). Early diagnosis of intestinal ischemia using urinary and plasma fatty acid binding proteins. Ann Surg.

[26] Vermeulen Windsant IC, Hellenthal FA, Derikx JP, Prins MH, Buurman WA, Jacobs MJ, Schurink GW (2012). Circulating intestinal fatty acid-binding protein as an early marker of intestinal necrosis after aortic surgery: a prospective observational cohort study. Ann Surg.

[27] Montagnana M, Danese E, Lippi G (2018). Biochemical markers of acute intestinal ischemia: possibilities and limitations. Ann Transl Med.

